# Gauging Functional Brain Activity: From Distinguishability to Accessibility

**DOI:** 10.3389/fphys.2019.00509

**Published:** 2019-05-08

**Authors:** David Papo

**Affiliations:** SCALab, UMR CNRS 9193, Université de Lille, Villeneuve d’Ascq, France

**Keywords:** functional brain activity, functional networks, spatial networks, structure, dynamics, geometry, topology, topological signal processing

## Abstract

Standard neuroimaging techniques provide non-invasive access not only to human brain anatomy but also to its physiology. The activity recorded with these techniques is generally called *functional* imaging, but what is observed *per se* is an instance of *dynamics*, from which functional brain activity should be extracted. Distinguishing between bare dynamics and genuine function is a highly non-trivial task, but a crucially important one when comparing experimental observations and interpreting their significance. Here we illustrate how neuroimaging’s ability to extract genuine functional brain activity is bounded by functional representations’ *structure*. To do so, we first provide a simple definition of functional brain activity from a system-level brain imaging perspective. We then review how the properties of the space on which brain activity is represented induce relations on observed imaging data which allow determining the extent to which two observations are functionally distinguishable and quantifying how far apart they are. It is also proposed that genuine functional distances would require defining *accessibility*, i.e., how a given observed condition can be accessed from another given one, under the dynamics of some neurophysiological process. We show how these properties result from the structure defined on dynamical data and dynamics-to-function projections, and consider some implications that the way and extent to which these are defined have for the interpretation of experimental data from standard system-level brain recording techniques.

## Introduction

System-level neuroimaging techniques such as PET and MRI make it possible to non-invasively access not only the anatomy of the human brain but also its physiology (Raichle, 2000). Brain activity recorded with these techniques, and others such as EEG or MEG is generally called *functional* imaging. However, observed activity is not genuinely functional *per se*, and neuroimaging data should *a priori* be treated as brain *dynamics*. Extracting functional brain activity from bare dynamics represents a non-trivial though often implicit process (Atmanspacher and Beim Graben, 2007; Allefeld et al., 2009).

Defining functional brain activity and how the brain implements given functions are arduous tasks. Here we address neither these ontological issues, nor the comparably complex one of state-space reconstruction from data, but a more circumscribed methodological question: how does neuroimaging data structure determine our ability to define functional activity?

Experimentalists typically compare representations associated with different recording sessions from the same individual, different individuals, or experimental conditions, addressing questions such as: when are two representations distinguishable? How far apart are they? What do neighboring representations look like? Is a transition possible from a given representation to another?

We illustrate how neuroimaging’s ability to address these questions is bounded by functional representations’ *structure*. We first provide a simple but convenient definition of functional brain activity from a system-level brain imaging perspective, a more comprehensive one being beyond the present work’s scope. We then review how the structure of the space on which brain activity is represented allows defining relations among observed instances of the dynamics, and show how these result from dynamics-to-function projections.

## Defining Functional Brain Activity

Function can be defined as the ability to perform a given cognitive or physiological task. Insofar as individuals’ behavioral performance results from brain properties, functional activity refers to both behavior and neural structures reflecting two complementary goals: understanding how brain anatomical structure and dynamics control function, and how task performance’s action produces functional brain subdivisions. In the former, a space Ψ of (typically non-observable) cognitive or physiological functions {ψ_1_, ψ_2_, ..., ψ_J_} is described using a finite set {φ_1_, φ_2_, ..., φ_K_} ∈ Φ_Obs_ of carefully selected coarse-grained aspects of brain anatomy or physiology (reflecting at a macroscopic level neurophysiological phenomena Φ_NObs_ not observable when using a given system-level neuroimaging technique) associated with observable performance measures {γ_1_, γ_2_, ..., γ_L_} ∈ Γ from subjects at rest or carrying out given tasks. In the latter, the ability to carry out given tasks is used as a probe exposing information on brain properties Φ.

Defining functional brain activity using system-level neuroimaging techniques involves partitioning two complex spaces, respectively made observable by behavior and brain recording techniques, putting some structure, i.e., a relationship among the set’s elements, on the set of equivalence classes, and mapping the corresponding structures.

## Brain Parcellation

Characterizing functional activity is in essence a parcellation problem. When using Φ to make sense of Ψ one ultimately aims at partitioning the space of cognitive functions *f* : Ψ → Ψ/ℜ where Ψ = Ψ(Γ, Φ) and Ψ/ℜ is the space of equivalence classes under the relation ℜ. In the opposite case, Φ is partitioned into functionally meaningful units *g* : Φ → Φ/ℜ′ using cognitive tasks as probes. This implies evaluating the sets *U* = π^−1^(V) where *U* ⊂ Ψ, *V* ⊂ Φ/ℜ′, π : Ψ → Φ/ℜ′ and ℜ′ is a relation defined on Φ or the equivalent in the opposite case. Since typically L ≪ K the structure on Φ is finer than that on Γ and physiology is more often used to define the cognitive space than the opposite case. Meaningful functional units correspond to the family of sets 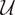
_π_ = {*U* = π^−1^(V)} (or, equivalently, 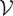
_π′_ = {V = π′^−1^(*U*)}). How to construct 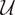
_π_ (or 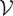
_π′_) what form the corresponding space may take, and therefore what may be regarded as functional, depends on the way (Ψ, 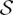
_Ψ_) and (Φ, 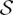
_Φ_) are defined and mapped onto each other through π (orπ′), 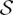 denoting a generic structure.

Classical neuropsychological descriptions map Γ onto the anatomical orthonormal Euclidean space (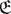; *d*) where *d* is the usual metric, so that Φ_
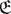
_ ⊂ ℝ^3^. Brain lesions induce a coarse partition Φ_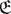/L_.
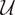
_π_ is extrapolated from the overlap between lesions and *cortical areas*, i.e., anatomical space partitions defined on the basis of cytoarchitecture, histological structure or organization homogeneity (Brodmann, 1909), associated with a map Ψ → Φ_
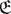
_, using double dissociations together with the assumption of modularity of both Φ_
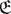
_ and Ψ (Dunn and Kirsner, 2003).

System-level neuroimaging maps Ψ onto some function of macroscopic observables φ_i_ ∈ Φ of brain physiology. Neuroimaging data are typically treated as (scalar, vector, or tensor) fields 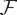 = {*f*_X_ (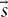, *t*)} where 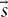 lives in a subspace isomorphic to ℝ^3^ and t ∈ ℝ is the physical time, and described in terms of some convenient function of this field, in the spatial (anatomical), temporal, frequency domains or in phase space, at experimental, developmental or evolutionary time-scales. On the other hand, while Γ is typically a scalar or vector field, it can sometimes take the form a complex *function space*.

Functional parcellations are defined in a recording technique- and scale-dependent manner. For fast sensory processes, functional equivalence classes can be defined by characterizing the dynamical *range*, i.e., the range of stimulus intensities resulting in distinguishable neural responses, while the dynamical *repertoire*, i.e., the number of distinguishable responses, quantifies the functional phase space extension. How to define distinguishability represents the most crucial question. For processes with non-trivial temporal scales such as thinking or reasoning (Papo, 2015) extracting function from dynamics is conceptually and technically arduous and involves understanding the structure of brain dynamics and how this can be used to ultimately define function.

## Superstructure of Brain Imaging Representations

The space on which parcellations are defined is in general endowed with some superstructure. First, brain anatomy and dynamics can be endowed with a network structure (Bullmore and Sporns, 2009), and, as a consequence, with topological properties (Boccaletti et al., 2006) and symmetries (Pecora et al., 2014). Network structures indicate that parcellations may not necessarily be local in the anatomical space.

Φ is typically embedded into (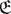; *d*), and treated as a field (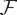; *d*) equipped with *d.* This translates the fact that, at least at the temporal scales at which anatomy represents a genuine boundary condition for brain anatomy and dynamics (Papo, 2017), the brain can be thought of as a spatial network (Barthélemy, 2011), submitted to geometric alongside topological constraints (Robinson P. A., 2013; Stiso and Bassett, 2018).

While Φ should not be regarded as homeomorphic to ℝ^n^, it may be treated as almost everywhere locally isomorphic to it, and represented as a *topological manifold* (*X*,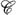) i.e., a *paracompact* topological space *X* equipped with an *atlas*, a cover 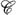 of open sets where each C ∈ 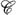 is homeomorphic to an open subset D ⊆ ℝ^n^ through a map φ_C_: C → D called a *chart* of 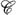 (Robinson M., 2013). Whenever data can effectively be treated as the output of a dynamical system, Φ may be modeled as a *topological dynamical system*, i.e., a triple (Φ, 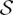, *T*_
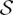
_) where Φ is a Hausdorff (separable) topological space, 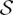 a topological semigroup prescribing the matching conditions between overlapping local trivialization charts, and T_
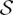
_ a continuous function T_
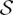
_: 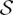
× Φ → Φ. For instance, long time scales fluctuations are characterized by non-trivial scaling properties such as scale-invariance (Novikov et al., 1997; Linkenkaer-Hansen et al., 2001; Allegrini et al., 2010; Expert et al., 2010; Papo, 2013), and the set of associated renormalization operators has a multiplicative semigroup structure on the time-scale space (Papo, 2014).

Φ can nonetheless be equipped with a geometry in various ways. First, geometry may be derived from topology. A network can always be embedded in a surface, provided it has sufficiently high genus (Aste et al., 2005); continuous space geometry may also emerge from the discrete network structure at microscopic scales, as in *pregeometric* models of quantum gravity (Bianconi and Rahmede, 2017). Furthermore, time series may be mapped into geometry, e.g., by representing observed brain activity in terms of probability distribution functions (Amari and Nagaoka, 2007; Lesne, 2014; Ali et al., 2018). This induces a smooth manifold 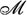 whose points are probability distributions defined on a common probability space (Amari and Nagaoka, 2007). Fluctuations’ scaling properties may help equipping the space with a specific geometry. For instance, scale-free distributions suggest a *fractal* geometry, for the temporal structure of spatially local fluctuations (Novikov et al., 1997; Linkenkaer-Hansen et al., 2001; Allegrini et al., 2010; Expert et al., 2010; Papo, 2013), but also for network representations of brain activity (Pasemann, 2002) whereas accounting for the history-dependence of brain fluctuations may require a *non-commutative* one or a quasi-metric space.

## Gauging Neuroimaging Data

Interpreting neuroimaging data requires introducing relations among experimental conditions and this, in turn, understanding the implications that given structures have on the definition of the families 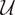
_π_ or, equivalently,
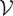
_π′_. Endowing data with given structural properties induces specific equivalence classes, e.g., two dynamical systems are dynamically equivalent if they are *topologically conjugate* (Xue and Bogdan, 2017). More generally, observed data may be classified up to a given property (e.g., homotopy, symmetry, etc) or by *obstructions* to one of them. Conversely, comparing experimental conditions involves comparing their associated (e.g., network) structure, each structure involving its own set of operations and restrictions, and sometimes adding further structure (Simas et al., 2015; Gadiyaram et al., 2016; Schieber et al., 2017).

At the most basic level, comparing experimental conditions requires evaluating the *topological distinguishability* of two sets *V*_1_ and *V*_2_ in φ/ℜ′ and the corresponding *U*_1_ and *U*_2_ in Ψ/ℜ. For the bare field representation, this requires comparing two fields *f*_X_ and *f*_Y_ a seemingly tractable task. However, noise, inter-individual differences and the possible organization of functional brain activity into patterns with similar meaning but considerably different anatomical structure (Ganmor et al., 2015) render distinguishability in terms of pattern similarity in (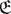; *d*) misleading.

The extent to which two parcellations can be distinguished depends on the space’s *separation* properties (Dodson and Parker, 1997). The functional space is not necessarily separable, even when Φ is embedded in (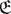; *d*). This is the case for fuzzy relations (Grzegorzewski, 2017) or overlapping communities (Palla et al., 2005) for which the manifold’s atlas charts overlap, and *transition functions* are needed to resolve these areas.

Observed data can be regarded as instances of an ensemble of objects with given properties, and equivalence class membership assessed using maximum entropy methods (Bianconi, 2007; Cimini et al., 2019). These properties’ meaningfulness can be gauged by their ability to perform a given task, e.g., classification or prediction (Zanin et al., 2016).

Often, it is also necessary to quantify how far *V*_1_ and *V*_2_ and the corresponding *U*_1_ and *U*_2_ are from each other. This implies defining some property intuitively translating the concept of *distance*. While the anatomically-embedded functional space can only locally be considered a Euclidean metric space, distances may be defined for other structures in a way that is dictated by the structure itself (Rossi et al., 2015; De Domenico and Biamonte, 2016). When operating in a probability distribution space, Φ can be equipped with the Fisher information metric e.g., by using the covariance matrix as a metric tensor (Crooks, 2007). This endows the space 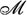 with a Riemannian differential manifold structure (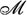, *g*, θ), *g* being the Fisher-Rao information metric and the parameters θ probability measures representing the manifold’s coordinates. The Fisher metric can be used to quantify the informational difference between measurements, and model predictions’ sensitivity to changes in parameters (Machta et al., 2013). Whenever neuroimaging data can be treated as a dynamical system, a dynamical distance can be derived from the dynamics itself. This distance allows a coarse-graining which in some sense is optimal with respect to the dynamics (Gaveau and Schulman, 2005). Finally, whether considering static or dynamic structures, perturbation methods can induce both a metric and *proximity* relations in Φ (Peters, 2016).

## From Dynamics to Function

To move from *dynamical* equivalence classes, comprising identical dynamical properties and symmetries, to *functional* equivalence classes, comprising patterns of neural activity that can achieve given functional properties (Ma et al., 2009) requires considering the structure induced by T_
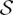
_Ψ__: Φ/Γ → Φ/Γ. While dynamical properties may sometimes be interpreted in functional terms, e.g., the co-existence of different attractors points to a given system’s multi-functionality (Xue and Bogdan, 2017), the dynamical system T_
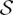
_Ψ__: Φ/Γ → Φ/Γ may give rise to non-trivial properties that cannot be anticipated based on dynamics alone.

**FIGURE 1:**
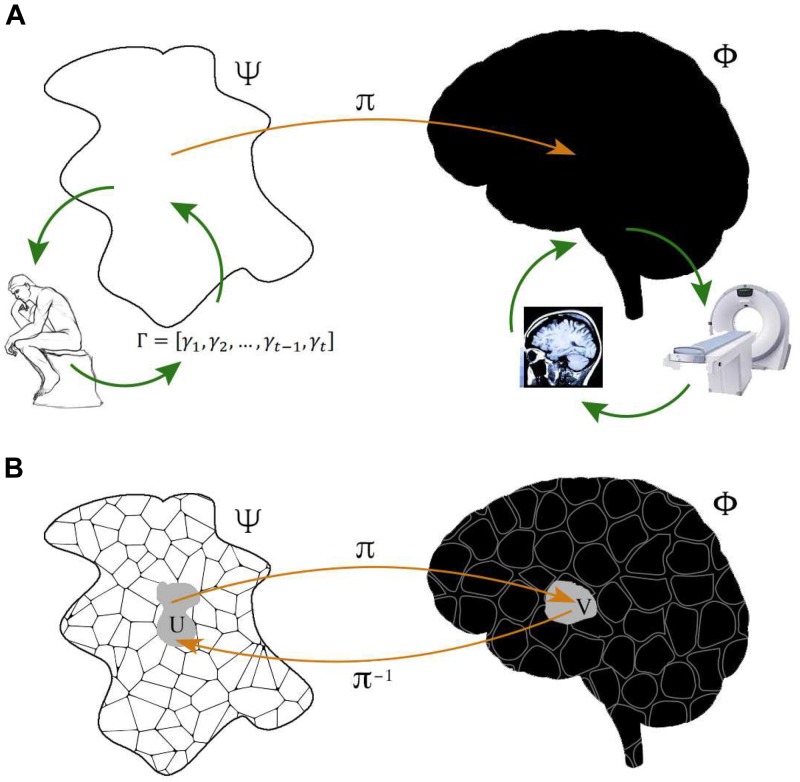
Representation of system-level functional brain mapping. **(A)** Typical neuroimaging experiments are designed to reconstruct a space Ψ of cognitive or physiological functions, made behaviorally observable by performance measures Γ from subjects at rest or carrying out given tasks, which can be associated, via some mapping π, with a space Φ defined by some brain property, observed through some neuroimaging technique. Experiments may also address the somehow dual aim of reconstructing Φ by using carefully selected cognitive of physiological probes, indirectly reflecting the space Ψ. Each neuroimaging technique documents specific neurophysiological phenomena Φ_Obs_ which result from brain activity Φ_NObs_ inaccessible to this particular technique. Note that functional brain activity Φ is typically identified with the anatomical space with Cartesian coordinates, but may in principle represent any other brain feature. **(B)** Φ and Ψ both have their own, usually non-trivial, structure i.e., a collection of elements together with a relation among them. The crucial point is that what is observed with a neuroimaging technique is not functional *per se*. Observed brain activity is genuinely functional if structure of the neurophysiological space Φ is reflected by structure in Ψ. Accordingly, neuroimaging’s goal is to use the structure of one space to refine the structure of the other, for all the elements of these spaces. The problem addressed by functional brain imaging can be thought of as a *fibe*r *bundle*, in essence a family of spaces (*fibers*) parameterized by another space (*base*). Such a structure can be used to define properties that are useful when gauging the significance of observed brain activity.

While each recording technique’s precision induces specific *a priori* parcellations of Φ, these are in general not functionally relevant. To be functionally meaningful, metrics in Φ need to be appraised in the space Ψ made observable through Γ. How properties in one structure are transferred onto those of the other depends on the map π. Ideally, one seeks the finest topology in Φ/ℜ′ that renders the π: Γ → Φ surjection continuous. This means endowing Φ with the *quotient topology* with respect to π i.e., the family *V*_π_ = {*V* |π^−1^(*V*) is open in Γ}. Thus, from a neuroimaging view-point, functional brain activity can be thought of as a fiber *bundle*, i.e., a quadruple (Ψ, Φ, π, 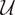
_π_), where Ψ is the total space, Φ the base π: Ψ → Φ a continuous surjective function called *projection*, and 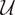
_π_ the fibers. Φ can be identified with a subspace of Ψ through a fiber bundle *section*, i.e., a continuous right inverse of the projection function π defined on open sets of Φ. Ψ is locally but not necessarily globally isomorphic to a Cartesian product Φ × 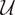
_π_ (see Figure 1).

Before examining the properties of the π: Ψ → Φ map, it is worth recalling that there exists a non-observable map 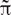: Φ_NObs_ → Φ_Obs_ which can show permutation symmetry but also combinatorial complexity with respect to more fine-grained Φ_NObs_ configurations (Brezina, 2010). A faithful representation of the hidden microscopic structure preserving given properties, e.g., symmetry (Cross and Gilmore, 2010), and the possibility to obtain a dynamical rule for the system (Allefeld et al., 2009) requires finding a generating partition, an arduous task in practice (Kantz and Schreiber, 2004). While macroscopic scale descriptions are *stricto sensu* dynamically emergent states only if they correspond to a Markov coarse-graining of lower-level dynamics (Adler, 1998; Shalizi and Moore, 2003; Bollt and Skufca, 2005; Gaveau and Schulman, 2005; Allefeld et al., 2009), both Φ_Obs_ and Γ can loosely be thought to emerge from the renormalization of microscopic neural fluctuations. How microscopic scales renormalize into macroscopic ones determines the scale at which the space is *locally isomorphic* to ℝ^n^ and can effectively be treated as a topological manifold. This scale may be induced by permutation symmetry with respect to a given property at microscopic scales. On the other hand, topologically equivalent structures may not have the same functional meaning in Φ_Obs_ and Φ_NObs_ For example, the robust computational properties associated with motifs in microcircuits (Klemm and Bornholdt, 2005; Gollo and Breakspear, 2014) do not necessarily characterize structurally isomorphic macroscopic circuits. Observability may also be increased by taking into account processes that are not directly observed when reconstructing the underlying dynamical system (Gupta et al., 2018).

The Φ_
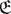
_ → Ψ map the lesion-based framework is in general ill-defined, due to fuzzy lesion contour geometry, and global non-Euclideanity but also to Φ′_
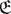
_*s* lack of temporal dimension and brain *degeneracy* (Price and Friston, 2002). However, Φ → Ψ maps can sometimes be well-behaved. A notable example is represented by Kelso’s bimanual finger coordination paradigm (Kelso, 1995). Once the relative phase *ϕ* between the fingers is chosen as the order parameter describing the dynamics, Φ and Ψ are both differentiable and Ψ turns out to be diffeomorphic to the macroscopic velocity field ∇ Φ, which in turn can be thought of as collective modes of underlying neurophysiological activity (Kelso et al., 1998). Since total space, base and fiber are all smooth differentiable manifolds and π is surjective, the functional space can be considered as a *differentiable fibe*r *bundle*. While in most contexts Ψ cannot be described in terms of differential equations or even dynamical rules, relatively well-behaved mappings may occur in other contexts as well. For instance, both brain networks (Meunier et al., 2010), and brain temporal fluctuations (Papo, 2014) display generic hierarchical structure which may be mirrored by one in Ψ, e.g., linguistic functions may be defined in terms of hierarchical relations, rules, and operations.

## From Measure to Accessibility

Proximity relations are usually quantified in terms of static representations, both for truly quasi-static data (e.g., fMRI images) and for dynamic ones (e.g., EEG recordings). However, these properties depend on the way one state in Φ/Γ may be transformed into another under some neurophysiological process.

To understand how the functional space inherits Φ′s properties, one may think of neurophysiological processes being only partially observable at the system level of non-invasive neuroimaging techniques, as *genotype*, and of observed behavior or macroscopic brain activity as the corresponding *phenotype*, resulting from coarse-graining of physiological processes. The crucial question is: what space does the genotype-to-phenotype map induce?

A smooth genotype-to-phenotype map can sometimes be ensured. For instance, in Kelso’s paradigm (Kelso et al., 1998), functional discontinuities in Ψ can be explained in terms of genuine brain dynamics. This results from the simultaneous fulfillment of various conditions: Φ′s *differentiable manifold* structure allows for differential calculus on the manifold; function is defined in terms of dynamical variables, i.e., synchronization and syncopation; components and collective variables in Ψ can both be endowed with explicit differentiable analytical expressions, and cognitive demands can be construed as their boundary conditions (Kelso, 1995).

**FIGURE 2:**
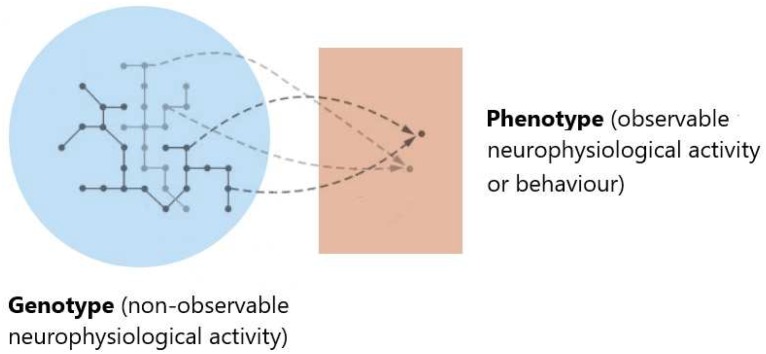
Gauging the functional space. Evaluating the significance of brain activity may require comparing data from different experimental conditions or subjects. This may involve not only asking whether given data sets are distinguishable, and quantifying how far apart they are but also establishing which configurations can be attained from given ones. Rather than the similarity of neuroimaging data representations, a dynamical definition of nearness and neighborhood should reflect the structure induced by the accessibility of given states induced by physiological process dynamics. This notion can be understood by thinking of Φ_NObs_ as *genotype*, and Φ_Obs_ (or observed behavior) as the corresponding *phenotype*. Characterizing accessibility in the phenotype space means understanding which phenotypic variations (corresponding to genuine functional differences) are realizable and which dynamical changes or image representation differences (depicted by networks of different gray shades in the genotype space) are functionally neutral, i.e., have equivalent function, with respect to underlying neurophysiological processes.

However, Ψ → Φ can induce non-trivial structure, and the phenotype space induced by T_
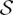
_Ψ__ may be non-metric, and even the less stringent notion of topology may not hold (Stadler et al., 2001; Stadler and Stadler, 2006). Nearness and neighborhood in the phenotype space should reflect the structure induced by genotype space’s *accessibility*, i.e., the ability to reach a given state *x* from another given state *y*, under the action of some underlying neurophysiological process: the variation operators establishing which configurations are accessible from given ones should reflect the dynamics of physiological processes (see Figure 2).

Ultimately, the phenotype’s properties depend on which variations are neurophysiologically neutral and which ones are realizable in the neighborhood of underlying neuronal variations. Since accessibility lacks symmetry in general, nearness in the induced space should be non-symmetric. Furthermore, dynamical patterns including intermittencies, degeneracy, and redundancy can result from the phenotype’s topological properties induced by the genotype-to-phenotype map.

## Concluding Remarks

Though not necessarily the case, some structures used in data analysis, viz. in topological data analysis, may reflect the way function impacts on brain dynamics. The brain can be thought of as a “geometric engine,” implementing structures (e.g., fiber bundles) through task-specific *functional architectures*, i.e., a hard-wired anatomical apparatus together with some dynamics (Koenderink and van Doorn, 1987), with non-random topological properties (Giusti et al., 2015; Curto et al., 2017), and (possibly non-Euclidean) geometry (Petitot, 2013, 2017). Moreover, representing brain data as probability distributions allows characterizing function as a perturbation of brain dynamics’ functional form, amplitude, or frequency modulations representing a short temporal scales special case (Papo, 2014). This representation induces a space of functions on Φ straightforwardly mirroring the non-observable space Ψ.

Topological signal processing tools are consistent with information locality (in space, time, and frequency, etc) (Robinson M., 2013). Likewise, despite novel techniques and high-performance computing, cortical areas are typically defined based on anatomically local structure-function associations (Amunts and Zilles, 2015), under the assumption that information is (locally) compact in 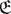. However, the brain also shows genuine non-locality, i.e., interaction-induced emergence, and the role of non-local information cannot be neglected (Santos et al., 2018).

Representing Φ as a space that does not derive its topology from a metric (Baruchi and Ben-Jacob, 2004; Petri et al., 2014) allows treating multiple observables, observation scales and geometries, and defining relationships between geometric objects constructed using different parameter values and continuous maps relating these objects (Carlsson, 2009).

Endowing Φ with a structure involves discretionary choices somehow associated with assumptions on what should be regarded as functional in brain activity, introducing circularity between definition and quantification of functional brain activity (Papo et al., 2014b). For instance, there are no criteria to elect the space to equip with a network structure, or to define its boundaries, constituent nodes and edges (Papo et al., 2014a). Brain function “stylized facts,” topological and geometrical constructs, and thorough behavioral studies (Krakauer et al., 2017) may all help defining and quantifying brain function. Finally, whether at the computational, algorithmic or implementation level (Marr, 1982), genuine mechanistic descriptions (Illari and Williamson, 2012) will help determining when the functional space can be endowed with a given representation and how reproducible it is.

## Data Availability

All datasets analyzed for this study are cited in the manuscript and the supplementary files.

## Author Contributions

DP thought and wrote the manuscript.

## Conflict of Interest Statement

The author declares that the research was conducted in the absence of any commercial or financial relationships that could be construed as a potential conflict of interest.

## References

[B1] Adler R. (1998). Symbolic dynamics and Markov partitions. *Bull. New Ser. Am. Math. Soc.* 35 1–56.

[B2] Ali S. A. Cafaro C. Gassner S. Giffin A. (2018). An information geometric perspective on the complexity of macroscopic predictions arising from incomplete information. *Adv. Math. Phys.* 2018:2048521.

[B3] Allefeld C. Atmanspacher H. Wackermann J. (2009). Mental states as macrostates emerging from electrical brain dynamics. *Chaos* 19:015102. 10.1063/1.3072788 19335006

[B4] Allegrini P. Menicucci D. Paradisi P. Gemignani A. (2010). Fractal complexity in spontaneous EEG metastable-state transitions: new vistas on integrated neural dynamics. *Front. Physiol.* 1:128. 10.3389/fphys.2010.00128 21423370 PMC3059954

[B5] Amari S. I. Nagaoka H. (2007). *Methods of Information Geometry* (Vol. 191). Providence, RI: American Mathematical Society.

[B6] Amunts K. Zilles K. (2015). Architectonic mapping of the brain beyond Brodmann. *Neuron* 88 1086–1107. 10.1016/j.neuron.2015.12.001 26687219

[B7] Aste T. Di Matteo T. Hyde S. T. (2005). Complex networks on hyperbolic surfaces. *Phys. A* 346 20–26.

[B8] Atmanspacher H. Beim Graben P. (2007). Contextual emergence of mental states from neurodynamics. *Chaos Complexity Lett.* 2:151.

[B9] Barthélemy M. (2011). Spatial networks. *Phys. Rep.* 499 1–101.

[B10] Baruchi I. Ben-Jacob E. (2004). Functional holography of recorded neuronal networks activity. *Neuroinformatics* 2 333–352. 15365195 10.1385/NI:2:3:333

[B11] Bianconi G. (2007). The entropy of randomized network ensembles. *Europhys. Lett.* 81:28005.

[B12] Bianconi G. Rahmede C. (2017). Emergent hyperbolic network geometry. *Sci. Rep.* 7:41974. 10.1038/srep41974 28167818 PMC5294422

[B13] Boccaletti S. Latora V. Moreno Y. Chavez M. Hwang D. U. (2006). Complex networks: structure and dynamics. *Phys. Rep.* 424 175–308.

[B14] Bollt E. M. Skufca J. D. (2005). “Markov partitions,” in *Encyclopedia of Nonlinear Science*, ed. Scott A. (New York, NY: Routledge).

[B15] Brezina V. (2010). Beyond the wiring diagram: signalling through complex neuromodulator networks. *Phil. Trans. R. Soc. B* 365 2363–2374. 10.1098/rstb.2010.0105 20603357 PMC2894954

[B16] Brodmann K. (1909). *Vergleichende Lokalisationslehre der Grosshirnrinde in Ihren Prinzipien Dargestellt auf Grund des Zellenbaues.* Leipzig: Barth JA.

[B17] Bullmore E. Sporns O. (2009). Complex brain networks: graph theoretical analysis of structural functional systems. *Nat. Rev. Neurosci.* 10 186–198. 10.1038/nrn2575 19190637

[B18] Carlsson G. (2009). Topology and data. *Bull. Am. Math. Soc.* 46 255–308.

[B19] Cimini G. Squartini T. Saracco F. Garlaschelli D. Gabrielli A. Caldarelli G. (2019). The statistical physics of real-world networks. *Nat. Rev. Phys.* 1 58–71.

[B20] Crooks G. E. (2007). Measuring thermodynamic length. *Phys. Rev. Lett.* 99:100602. 10.1103/PhysRevLett.99.100602 17930381

[B21] Cross D. J. Gilmore R. (2010). Differential embedding of the Lorenz attractor. *Phys. Rev. E* 81:066220. 20866514 10.1103/PhysRevE.81.066220

[B22] Curto C. Gross E. Jeffries J. Morrison K. Omar M. Rosen Z. (2017). What makes a neural code convex? *SIAM J. Appl. Algebra Geom.* 1 222–238.

[B23] De Domenico M. Biamonte J. (2016). Spectral entropies as information-theoretic tools for complex network comparison. *Phys. Rev. X* 6:041062.

[B24] Dodson C. T. Parker P. E. (1997). *A User’s Guide to Algebraic Topology*, Vol. 387 Berlin: Springer Science & Business Media.

[B25] Dunn J. C. Kirsner K. (2003). What can we infer from double dissociations? *Cortex* 39 1–7. 12627749 10.1016/s0010-9452(08)70070-4

[B26] Expert P. Lambiotte R. Chialvo D. R. Christensen K. Jensen H. J. Sharp D. J. (2010). Self-similar correlation function in brain resting-state functional magnetic resonance imaging. *J. R. Soc. Interface* 8 472–479. 10.1098/rsif.2010.0416 20861038 PMC3061122

[B27] Gadiyaram V. Ghosh S. Vishveshwara S. (2016). A graph spectral-based scoring scheme for network comparison. *J. Complex Netw.* 5 219–244. 10.1002/prot.25332 28598579

[B28] Ganmor E. Segev R. Schneidman E. (2015). A thesaurus for a neural population code. *eLife* 4:e06134. 10.7554/eLife.06134 26347983 PMC4562117

[B29] Gaveau B. Schulman L. S. (2005). Dynamical distance: coarse grains, pattern recognition, and network analysis. *Bull. Sci. Math.* 129 631–642.

[B30] Giusti C. Pastalkova E. Curto C. Itskov V. (2015). Clique topology reveals intrinsic geometric structure in neural correlations. *Proc. Natl. Acad. Sci. U.S.A.* 112 13455–13460. 10.1073/pnas.1506407112 26487684 PMC4640785

[B31] Gollo L. L. Breakspear M. (2014). The frustrated brain: from dynamics on motifs to communities and networks. *Phil. Trans. R. Soc. B* 369:20130532. 10.1098/rstb.2013.0532 25180310 PMC4150307

[B32] Grzegorzewski P. (2017). On separability of fuzzy relations. *Int. J. Fuzzy Log. Intell. Syst.* 17 137–144.

[B33] Gupta G. Pequito S. Bogdan P. (2018). “Dealing with unknown unknowns: identification and selection of minimal sensing for fractional dynamics with unknown inputs,” in *Proceedings of the 2018 Annual American Control Conference (ACC)*, (Milwaukee, WI: IEEE), 2814–2820.

[B34] Illari P. M. Williamson J. (2012). What is a mechanism? Thinking about mechanisms across the sciences. *Eur. J. Philos. Sci.* 2 119–135.

[B35] Kantz H. Schreiber T. (2004). *Nonlinear Time Series Analysis*, Vol. 7 Cambridge: Cambridge university press.

[B36] Kelso J. A. S. (1995). *Dynamic Patterns: the Self-Organization of Brain and Behavior.* Cambridge, MA: MIT Press.

[B37] Kelso J. A. S. Fuchs A. Holroyd T. Lancaster R. Cheyne D. Weinberg H. (1998). Dynamic cortical activity in the human brain reveals motor equivalence. *Nature* 392 814–818. 9572140 10.1038/33922

[B38] Klemm K. Bornholdt S. (2005). Topology of biological networks and reliability of information processing. *Proc. Natl. Acad. Sci. U.S.A.* 102 18414–18419. 16339314 10.1073/pnas.0509132102 PMC1317958

[B39] Koenderink J. J. van Doorn A. J. (1987). Representation of local geometry in the visual system. *Biol. Cybern.* 55 367–375. 3567240 10.1007/BF00318371

[B40] Krakauer J. W. Ghazanfar A. A. Gomez-Marin A. MacIver M. A. Poeppel D. (2017). Neuroscience needs behavior: correcting a reductionist bias. *Neuron* 93 480–490. 10.1016/j.neuron.2016.12.041 28182904

[B41] Lesne A. (2014). Shannon entropy: a rigorous notion at the crossroads between probability, information theory, dynamical systems and statistical physics. *Math. Struct. Comput. Sci.* 24:e240311.

[B42] Linkenkaer-Hansen K. Nikouline V. V. Palva J. M. Ilmoniemi R. (2001). Long-range temporal correlations and scaling behavior in human oscillations. *J. Neurosci.* 15 1370–1377. 10.1523/JNEUROSCI.21-04-01370.2001 PMC6762238 11160408

[B43] Ma W. Trusina A. El-Samad H. Lim W. A. Tang C. (2009). Defining network topologies that can achieve biochemical adaptation. *Cell* 138 760–773. 10.1016/j.cell.2009.06.013 19703401 PMC3068210

[B44] Machta B. B. Chachra R. Transtrum M. K. Sethna J. P. (2013). Parameter space compression underlies emergent theories and predictive models. *Science* 342 604–607. 10.1126/science.1238723 24179222

[B45] Marr D. (1982). *Vision: a Computational Approach.* San Francisco, CA: Freeman & Co.

[B46] Meunier D. Lambiotte R. Bullmore E. T. (2010). Modular and hierarchically modular organization of brain networks. *Front. Neurosci.* 4:200 10.3389/fnins.2010.00200 PMC3000003 21151783

[B47] Novikov E. Novikov A. Shannahoff-Khalsa D. Schwartz B. Wright J. (1997). Scale-similar activity in the brain. *Phys. Rev. E* 56 R2387–R2389.

[B48] Palla G. Derényi I. Farkas I. Vicsek T. (2005). Uncovering the overlapping community structure of complex networks in nature and society. *Nature* 435 814–818. 15944704 10.1038/nature03607

[B49] Papo D. (2013). Time scales in cognitive neuroscience. *Front. Physiol.* 4:86 10.3389/fphys.2013.00086 PMC3630296 23626578

[B50] Papo D. (2014). Functional significance of complex fluctuations in brain activity: from resting state to cognitive neuroscience. *Front. Syst. Neurosci.* 8:112. 10.3389/fnsys.2014.00112 24966818 PMC4052734

[B51] Papo D. (2015). How can we study reasoning in the brain? *Front. Hum. Neurosci.* 9:222. 10.3389/fnhum.2015.00222 25964755 PMC4408754

[B52] Papo D. (2017). Beyond the anatomy-based representation of brain function. Comment on “Topodynamics of metastable brains” by Arturo Tozzi et al. *Phys. Life Rev.* 21 42–45. 28460822 10.1016/j.plrev.2017.04.005

[B53] Papo D. Zanin M. Buldú J. M. (2014a). Reconstructing functional brain networks: have we got the basics right? *Front. Hum. Neurosci.* 8:107. 24578687 10.3389/fnhum.2014.00107 PMC3936558

[B54] Papo D. Zanin M. Pineda J. A. Boccaletti S. Buldú J. M. (2014b). Brain networks: great expectations, hard times, and the big leap forward. *Phil. Trans. R. Soc. B* 369:20130525. 10.1098/rstb.2013.0525 25180303 PMC4150300

[B55] Pasemann F. (2002). Complex dynamics and the structure of small neural networks. *Netw. Comput. Neural Syst.* 13 195–216. 12061420

[B56] Pecora L. M. Sorrentino F. Hagerstrom A. M. Murphy T. E. Roy R. (2014). Cluster synchronization and isolated desynchronization in complex networks with symmetries. *Nat. Commun.* 5:4079. 10.1038/ncomms5079 24923317

[B57] Peters J. F. (2016). *Computational Proximity. Intelligent Systems Reference Library*, Vol. 102 Cham: Springer.

[B58] Petitot J. (2013). Neurogeometry of neural functional architectures. *Chaos Soliton. Fract.* 50 75–92. 10.3389/neuro.04.001.2008 18946541 PMC2526276

[B59] Petitot J. (2017). *Elements of Neurogeometry: Functional Architectures of Vision.* Berlin: Springer.

[B60] Petri G. Expert P. Turkheimer F. Carhart-Harris R. Nutt D. Hellier P. J. (2014). Homological scaffolds of brain functional networks. *J. R. Soc. Interface* 11:20140873. 10.1098/rsif.2014.0873 PMC4223908 25401177

[B61] Price C. J. Friston K. J. (2002). Degeneracy and cognitive anatomy. *Trends Cogn. Sci.* 6 416–421. 12413574 10.1016/s1364-6613(02)01976-9

[B62] Raichle M. E. (2000). Functional brain imaging and human brain function. *J. Neurosci.* 23 3959–3962. 10.1523/JNEUROSCI.23-10-03959.2003 PMC6741098 12764079

[B63] Robinson M. (2013). *Topological Signal Processing.* Berlin: Springer.

[B64] Robinson P. A. (2013). Discrete-network versus modal representations of brain activity: why a sparse regions-of-interest approach can work for analysis of continuous dynamics. *Phys. Rev. E* 88:054702. 24329391 10.1103/PhysRevE.88.054702

[B65] Rossi L. Torsello A. Hancock E. R. (2015). Measuring graph similarity through continuous-time quantum walks and the quantum Jensen-Shannon divergence. *Phys. Rev. E* 91:022815. 25768560 10.1103/PhysRevE.91.022815

[B66] Santos F. A. N. Raposo E. P. Coutinho-Filho M. D. Copelli M. Stam C. J. Douw L. (2018). Topological phase transitions in functional brain networks. *bioRxiv* [Preprint]. 10.1101/469478 31640025

[B67] Schieber T. A. Carpi L. Díaz-Guilera A. Pardalos P. M. Masoller C. Ravetti M. G. (2017). Quantification of network structural dissimilarities. *Nat. Commun* 8:13928. 10.1038/ncomms13928 28067266 PMC5227707

[B68] Shalizi C. R. Moore C. (2003). What is a macrostate? Subjective observations and objective dynamics. arXiv:cond-mat/0303625 [Preprint].

[B69] Simas T. Chavez M. Rodriguez P. R. Diaz-Guilera A. (2015). An algebraic topological method for multimodal brain networks comparisons. *Front. Psychol.* 6:904. 10.3389/fpsyg.2015.00904 26217258 PMC4491601

[B70] Stadler B. M. R. Stadler P. F. Wagner G. P. Fontana W. (2001). The topology of the possible: formal spaces underlying patterns of evolutionary change. *J. Theor. Biol.* 213 241–274. 11894994 10.1006/jtbi.2001.2423

[B71] Stadler P. F. Stadler B. M. (2006). Genotype-phenotype maps. *Biol. Theor.* 1 268–279.

[B72] Stiso J. Bassett D. S. (2018). Spatial embedding imposes constraints on neuronal network architectures. *Trends Cogn. Neurosci.* 22 1127–1142. 30449318 10.1016/j.tics.2018.09.007

[B73] Xue Y. Bogdan P. (2017). Reliable multi-fractal characterization of weighted complex networks: algorithms and implications. *Sci. Rep.* 7:7487. 10.1038/s41598-017-07209-5 28790321 PMC5548933

[B74] Zanin M. Papo D. Sousa P. A. Menasalvas E. Nicchi A. Kubik E. (2016). Combining complex networks and data mining: why and how. *Phys. Rep.* 635 1–44.

